# Near-infrared quantum cutting in Ho^3+^, Yb^3+^-codoped BaGdF_5_ nanoparticles via first- and second-order energy transfers

**DOI:** 10.1186/1556-276X-7-636

**Published:** 2012-11-22

**Authors:** Linna Guo, Yuhua Wang, Jia Zhang, Yanzhao Wang, Pengyu Dong

**Affiliations:** 1 Department of Materials Science, School of Physical Science and Technology, Lanzhou University, Lanzhou, 730000, People's Republic of China

**Keywords:** Near infrared quantum cutting, First- and second-order energy transfers, Back energy transfer, BaGdF_5_: Ho^3+^, Yb^3+^

## Abstract

Infrared quantum cutting involving Yb^3+^ 950–1,000 nm (^2^ F_5/2_ → ^2^ F_7/2_) and Ho^3+^ 1,007 nm (^5^S_2_,^5^F_4_ → ^5^I_6_) as well as 1,180 nm (^5^I_6_ → ^5^I_8_) emissions is achieved in BaGdF_5_: Ho^3+^, Yb^3+^ nanoparticles which are synthesized by a facile hydrothermal route. The mechanisms through first- and second-order energy transfers were analyzed by the dependence of Yb^3+^ doping concentration on the visible and infrared emissions, decay lifetime curves of the ^5^ F_5_ → ^5^I_8_, ^5^S_2_/^5^F_4_ → ^5^I_8_, and ^5^ F_3_ → ^5^I_8_ of Ho^3+^, in which a back energy transfer from Yb^3+^ to Ho^3+^ is first proposed to interpret the spectral characteristics. A modified calculation equation for quantum efficiency of Yb^3+^-Ho^3+^ couple by exciting at 450 nm was presented according to the quantum cutting mechanism. Overall, the excellent luminescence properties of BaGdF_5_: Ho^3+^, Yb^3+^ near-infrared quantum cutting nanoparticles could explore an interesting approach to maximize the performance of solar cells.

## Background

Lanthanide (Ln) ions could exhibit both efficient upconversion (UC) and downconversion (DC) emission properties [1], where the UC process converts low-energy light, usually near infrared (NIR) or infrared, to higher energies, ultraviolet or visible, via multiple absorptions or energy transfers (ETs). In contrast, DC process is the conversion of higher-energy photons into lower-energy photons [2]. For the time being, DC of NIR luminescence (i.e., NIR quantum cutting (QC)), which down-converts one incident UV-blue photon into two NIR photons (approximately 1,000 nm), has attracted more attention for their application in silicon solar cells by modifying the incident light wavelength [3].

As it is well known to us that the solar spectrum and the bandgap energy of silicon semiconductor do not match each other, thus photons with energy lower than the bandgap could not be absorbed, while for photons with energy larger than the bandgap, the excess energy is lost by thermalization of hot charge carriers [4]. Take these sources of energy loss into account for the solar spectrum; the maximum energy efficiency is 30% only for a crystalline Si solar cell with a bandgap of 1.12 eV [3]. Considering this, if the conversion of one UV or visible photon into two NIR photons with energies about 1.12 eV is realized through QC in a silicon solar cell, the energy loss related to thermalization of hot charge carriers can be reduced, and the solar cell efficiency will be enhanced greatly to satisfy the application requirement [5].

To obtain high NIR QC efficiency, other Ln^3+^ ions are generally codoped with Yb^3+^ in the hosts, and it is commonly demonstrated in Ln^3+^-Yb^3+^ (Ln = Tb, Tm, Pr, Er, Nd, and Ho) couple-codoped materials [6]. Generally speaking, there are two mechanisms involved in the NIR QC in Ln^3+^-Yb^3+^ couple [5]: one is second-order cooperation of energy transfer (CET) based on one donor and two acceptor ions, and the other is first-order resonance energy transfer (ET). Nevertheless, CET process is not as efficient as resonance ET [7-9], but high CET efficiency would be realized at high Yb^3+^ concentration. Thus, a NIR QC via resonant first-order ET process seems more favorable for luminescent materials, which requires an intermediate energy level of donor ion to resonantly excite acceptor ions by a two-step ET process. There are many reports about second-order CET [2,10-14]; however, there are few reports about first- and second-order ETs occurring simultaneously in one research system.

On the other hand, to fulfill the requirements of NIR QC, host materials should have the energy of phonons as low as possible in order to reduce probabilities of multiphonon relaxations between spaced energy levels of Ln^3+^ ions. Cubic BaGdF_5_, a tri-fluoride compound, has a wide bandgap and low phonon energy which is a suitable NIR QC matrix [15]. Meanwhile, Ho^3+^ ion has favorable metastable energy levels and considerable energy match between Yb^3+^ and Ho^3+^, so Ho^3+^/Yb^3+^ couple could be a good choice in NIR QC investigation. However, NIR QC reports in Ho^3+^/Yb^3+^-codoped materials are limited in NaYF_4_ and glass ceramic [7,9]. In addition, considering NIR nanomaterials is convenient in the practical application of the coating for the solar cells. Herein, we prepared BaGdF_5_: Ho^3+^, Yb^3+^ nanoparticles with different Yb^3+^ concentrations by a trisodium citrate (Cit^3−^)-assisted hydrothermal method, which is less finicky, low-cost, and effective for large-scale production. Furthermore, NIR QC via first- and second-order resonant ET processes and a back ET from Yb^3+^ to Ho^3+^ in BaGdF_5_: Ho^3+^, Yb^3+^ nanoparticles is firstly investigated, and the corresponding quantum efficiencies (QE) are also calculated.

## Methods

BaGd_1 − 0.01 − *x*%_Yb_*x*%_Ho_0.01_ F_5_ (0 ≤ *x* ≤ 20) samples were prepared by a hydrothermal process. Firstly, 1 mmol rare earth oxides Gd_2_O_3_, Yb_2_O_3_, and Ho_2_O_3_ were dissolved in dilute HNO_3_ solution, and the residual HNO_3_ was removed by heating and evaporation, resulting in the formation of clear solution of RE(NO_3_)_3_ (RE = Gd, Yb, Ho). Cit^3−^ aqueous solution was added into the Ba(NO_3_)_2_·2H_2_O and RE(NO_3_)_3_ solution to form metal-Cit complex. After vigorous stirring for 30 min, aqueous solution containing 4 mmol NaBF_4_ was poured into the above solution, and pH value of the mixture was adjusted to about 4.5 by adding diluted HCl or NH_3_·H_2_O. After additional agitation for 15 min, the as-obtained mixed solution was transferred into a 50-ml teflon autoclave, which was tightly sealed and maintained at 180°C for 24 h. As the autoclave was cooled to room temperature naturally, the resulting precipitates were separated via centrifugation, dried in oven at 80°C for 12 h.

### Characterizaton

The XRD patterns were obtained on Rigaku D/max-2400 powder diffractometer (Rigaku Corporation, Tokyo, Japan) using Cu Kα radiation (1.5405 Å) at 40 kV and 60 mA. The size, shape, and structure of the as-prepared samples were characterized by SEM (S-4800). Emission and excitation measurements were performed using an Edinburgh Instruments' FLS920 fluorescence spectrometer (Livingston, UK), and a 0.3-m double excitation monochromator and two emission monochromators to record the emission spectra in the wavelength range of 200–850 nm (with a Hamamatsu R928 photomultiplier tube, Bridgewater, NJ, USA) or in the wavelength range of 850–1650 nm (with a liquid nitrogen-cooled Hamamatsu R5509-72 PMT). All spectra were measured at room temperature.

## Results and discussion

**Figure 1:**
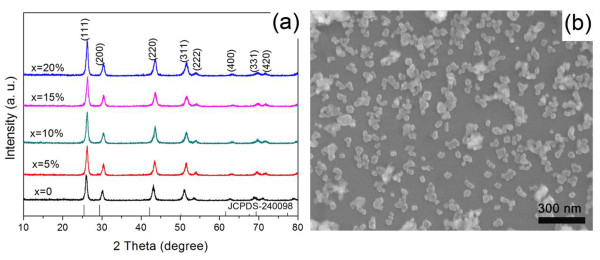
**XRD patterns and SEM image.** (**a**) The measured XRD patterns of BaGdF_5_: 1%Ho^3+^, *x*%Yb^3+^ (0 ≤ *x* ≤ 20) as well as the standard XRD pattern of BaGdF_5_ (JCPDS no. 24–0098) used as a reference. (**b**) SEM image of BaGdF_5_: 1%Ho^3+^ nanoparticle as a representative.

The XRD patterns and representative SEM photograph of BaGd_1 − 0.01 − *x*%_Yb_*x*%_Ho_0.01_ F_5_ (0 ≤ *x* ≤ 20) nanoparticles are shown in Figure 1a, b, respectively. It is obvious that the locations and relative intensities of the diffraction peaks coincide well with the data reported in the JCPDS standard card (no. 24–0098). No additional peaks of other phases were found, indicating that the pure-phase BaGdF_5_ is obtained. It can be seen from Figure 1b that the nanoparticles are relatively dispersed with uniform granular morphology and sizes which are about 40 nm.

**Figure 2:**
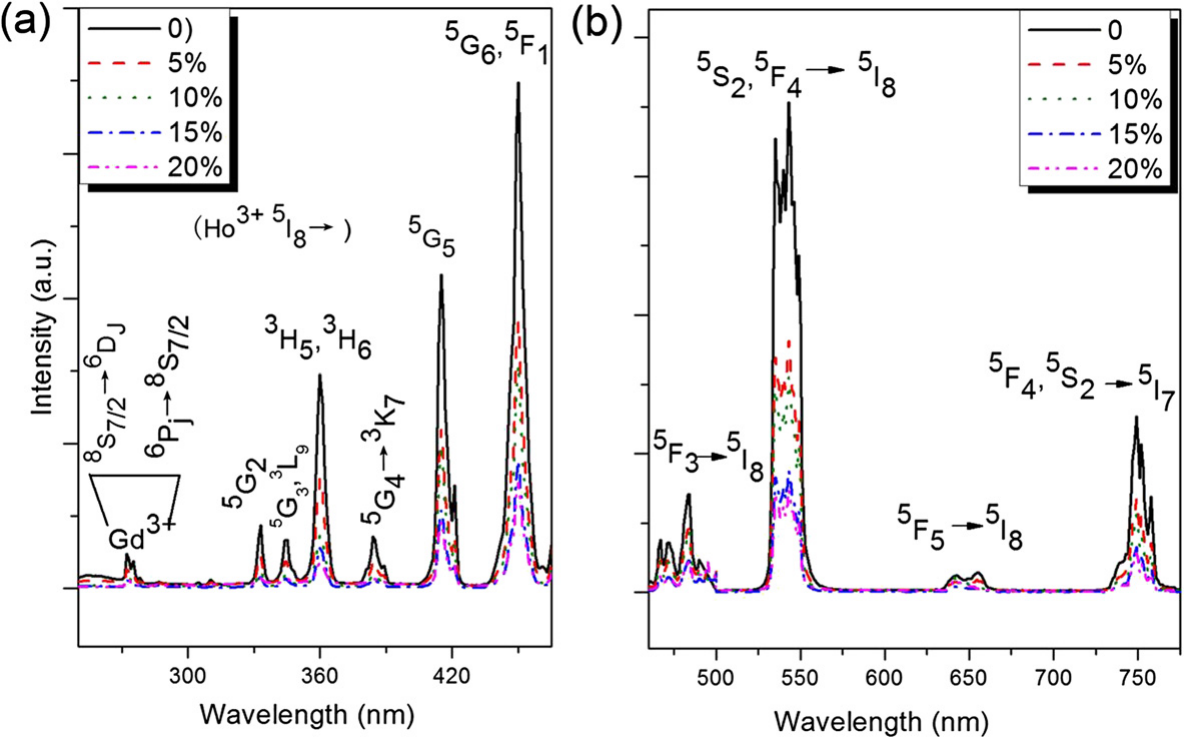
**PLE and PL spectra by monitoring Ho**^**3+**^**emission.** (**a**) PLE spectra by monitoring Ho^3+^: ^5^S_2_ → ^5^I_8_ emission at 540 nm and (**b**) visible PL spectra under excitation of 450 nm (^5^I_8_ → ^5^ G_6_, ^5^ F_1_) of BaGdF_5_: 1% Ho^3+^, *x*% Yb^3+^ (0 ≤ *x* ≤ 20) nanoparticles.

**Figure 3:**
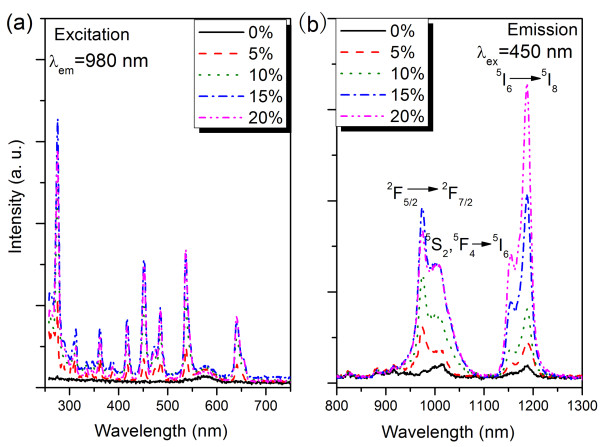
**PLE and PL spectra monitoring of the Yb**^**3+**^**emission.** (**a**) PLE spectra monitoring of the Yb^3+^: ^2^ F_5/2_ → ^2^ F_7/2_ emission and (**b**) PL spectra in the infrared region under excitation of 450 nm (^5^I_8_ → ^5^ G_6_, ^5^ F_1_) of BaGdF_5_: 1% Ho^3+^, *x*% Yb^3+^ (0 ≤ *x* ≤ 20) nanoparticles.

Photoluminescence excitation (PLE) spectra monitoring at 540 nm and photoluminescence (PL) spectra in the visible region under 450-nm excitation of BaGdF_5_: 1% Ho^3+^, *x*% Yb^3+^ (0 ≤ *x* ≤ 20) nanoparticles were investigated, as shown in Figure 2a,b, respectively. It is noticed from Figure 2a that the most intense excitation band of Ho^3+^ is at 450 nm, corresponding to the ^5^I_8_ → ^5^ G_6_, ^5^ F_1_ transition of Ho^3+^ ions. Figure 2b shows the emission peaks of Ho^3+^ at about 483, 545, 651, and 747 nm which are attributed to the ^5^ F_3_ → ^5^I_8_, ^5^ F_4_/^5^S_2_ → ^5^I_8_, ^5^ F_5_ → ^5^I_8_, and ^5^S_2_ → ^5^I_7_ transitions of Ho^3+^, respectively [16,17]. It is worthwhile pointing out that both the excitation and emission intensities decrease with increasing Yb^3+^ concentration, which may be due to the Ho^3+^ transferring its energy to the Yb^3+^, but it needs more evidence to testify this guess, which will be discussed as follows:

PLE spectra of BaGdF_5_: 1% Ho^3+^, *x*% Yb^3+^ (0 ≤ *x* ≤ 20) nanoparticles are also recorded by monitoring the characteristic emission of Yb^3+^ at 980 nm (Figure 3a). The presence of Ho^3+^ excitation in the PLE spectra of BaGdF_5_: 1% Ho^3+^, *x*% Yb^3+^ by monitoring the characteristic emission of Yb^3+^ gives an evidence to the energy transfer from Ho^3+^ to Yb^3+^. Furthermore, it is noteworthy that a broad emission band in the range of 950–1,100 nm, corresponding to the ^2^ F_5/2_ → ^2^ F_7/2_ transition of Yb^3+^ and ^5^S_2_,^5^F_4_ → ^5^I_6_ transition of Ho^3+^ ions, has also been observed under the Ho^3+^ excitation at 450 nm (as shown in Figure 3b). This is another evidence of ET from Ho^3+^ to Yb^3+^. Simultaneously, the 1,180-nm emission due to ^5^I_6_ → ^5^I_8_ transition of Ho^3+^ is also detected. NIR emission intensity of Yb^3+^ intensifies rapidly with increasing Yb^3+^ concentration from 0 to 15 mol%. Whereas, 1,180-nm emission intensity of Ho^3+^ enhances with increasing Yb^3+^ concentration even when Yb^3+^ concentration is 20 mol%. That is to say, the NIR emission quenching concentration of Ho^3+^ is higher than that of Yb^3+^. The reasons for the above phenomenon will be discussed later.

**Figure 4:**
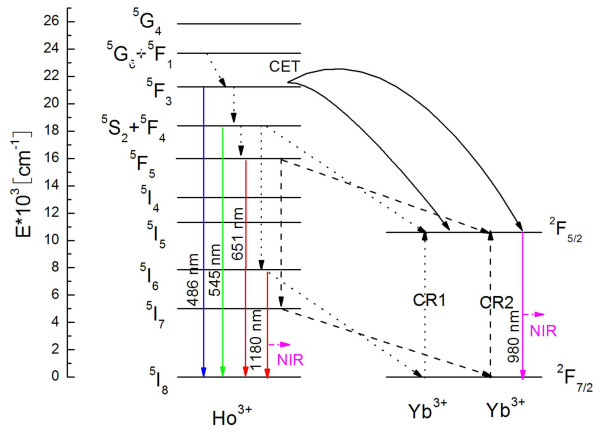
**Energy level diagram of Ho**^**3+**^**and Yb**^**3+**^**showing possible mechanisms for a NIR QC processes.**

For excitation at 450 nm, there are three possible ET processes from Ho^3+^ to Yb^3+^ responsible for the NIR emission of Yb^3+^ ions, in view of energy levels of Ho^3+^ xand Yb^3+^ ions (shown in Figure 4) [18,19]. (1) Ho^3+^: ^5^ F_3_ → ^5^I_8_ transition is located at approximately twice the energy of the Yb^3+^: ^2^ F_5/2_ → ^2^ F_7/2_ transition, which in theory can transfer its energy to Yb^3+^ ions through a CET mechanism: Ho^3+^(^5^ F_3_) → 2Yb^3+^(^2^ F_5/2_) + *hν*; (2) Ho^3+^ ion could relax from its ^5^S_2_, ^5^ F_4_ to ^5^I_6_ level and then transfer its energy to only one Yb^3+^ ion, which belongs to first-order resonance CR1 process (^5^S_2_, ^5^ F_4_(Ho) + ^2^ F_7/2_(Yb) → ^5^I_6_(Ho) + ^2^ F_7/2_(Yb) + *hν*; and (3) similarly, CR2 process: ^5^ F_5_(Ho) + ^2^ F_7/2_(Yb) → ^5^I_7_(Ho) + ^2^ F_7/2_ (Yb) + *hν*.

**Figure 5:**
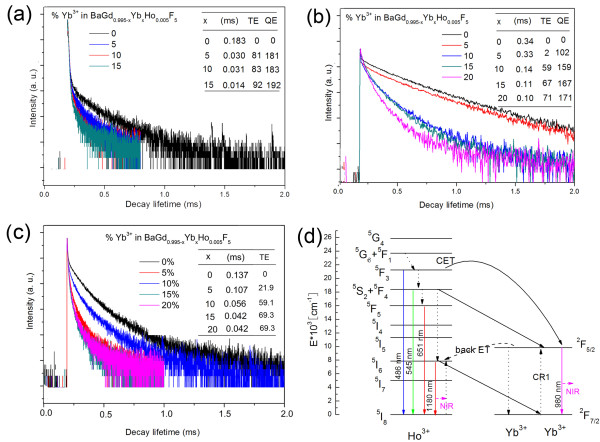
**Decay curves of Ho**^**3+**^**.** For (**a**) ^5^ F_3_ → ^5^I_8_, (**b**) ^5^ F_4_, ^5^S_2_ → ^5^I_8_, and (**c**) ^5^ F_5_ → ^5^I_8_ emissions under excitation of 450 nm. The inset shows the average decay lifetime and energy transfer efficiency as a function of Yb^3+^ doping concentration. (**d**) Energy level diagram of Ho^3+^ and Yb^3+^ showing mechanisms for NIR QC processes.

According to the above mechanisms, the emission of Ho^3+^: ^5^I_6_ → ^5^I_8_ is mainly followed by the occurrence of CR1 process. Also, as it is well known to us that CET process is not efficient at lower Yb^3+^ content due to its intrinsic nature, but at a relatively high Yb^3+^ concentration of 15 mol%, the CET process could become efficient, and this efficiency (*η*_CET_) can be estimated by Equation 1: [11]

(1) $$\eta_{\text{ET}}=\eta_{x\%\text{Yb}}=1-\frac{\int I_{x\%\text{Yb}}dt}{\int I_{0\%\text{Yb}}dt}$$

where *I* denotes the decay intensity, and *x*% Yb denotes the Yb^3+^ contents. To determine the *η*_CET_ for BaGdF_5_: 1% Ho^3+^, *x*% Yb^3+^ (0 ≤ *x* ≤ 15) nanoparticles, a series of decay curves of Ho^3+^: ^5^ F_3_ → ^5^I_8_ emissions at 486 nm are determined, as shown in Figure 5a. All the decay curves demonstrate double-exponential feature (Table 1), so the decay times can be determined using a curve fitting technique based on the following equation:

(2) $$I=A_{1}\text{exp}\left(-\frac{t}{\tau_{1}}\right)+A_{2}\text{exp}\left(-\frac{t}{\tau_{2}}\right)$$

where *I* is phosphorescence intensity; *A*_1_ and *A*_2_, constants; *t*, time; and *τ*_1_ and *τ*_2_, decay constants deciding the rates for the rapid and the slow exponentially decay components, respectively. The average decay times (*τ*) can be calculated by the following formula:

(3) $$<\tau >=\left(A_{1}\tau_{1}^{2}+A_{2}\tau_{2}^{2}\right)/\left(A_{1}\tau_{1}+A_{2}\tau_{2}\right)$$

**Table 1:** The fitting results of parameters of the double-exponential decays

**Monitoring wavelength**	**Yb**^**3+**^**concentration (mol%)**	***τ***_**1**_**(μs)**	***A***_**1**_	***τ***_**2**_**(μs)**	***A***_**2**_	***X***^**2**^
486 nm (^5^ F_3_ → ^5^I_8_)	0	23.0494	127.162	204.1946	110.036	1.493
	5	6.2807	1,731.739	68.1584	117.146	1.521
	10	6.1677	4,114.752	82.2261	152.536	1.397
	15	5.0831	4,341.002	49.8707	105.941	1.558
	20	5.1521	1,799.106	34.5770	74.643	1.500
545 nm (^5^S_2_ → ^5^I_8_)	0	143.0003	2,221.670	399.7964	2,634.467	1.473
	5	106.2121	1,454.757	378.4078	2,065.503	1.359
	10	60.6316	2,270.273	199.2826	885.756	1.612
	15	50.8624	2,892.493	166.1588	1,010.801	1.539
	20	52.5225	666.286	152.9727	282.404	1.484
651 nm (^5^ F_5_ → ^5^I_8_)	0	68.2479	1,422.916	206.0216	472.991	1.421
	5	38.4695	903.197	157.5332	307.477	1.576
	10	18.9739	650.965	87.4463	170.137	1.496
	15	14.1099	691.857	71.4125	126.626	1.528
	20	138.514	669.904	67.1548	151.641	1.416

Also, the corresponding lifetime values as well as *η*_CET_ are calculated, which are summarized in the inset of Figure 5a. Unexpectedly, *η*_CET_ is calculated to be as high as 92% in the BaGdF_5_: 1% Ho^3+^, 15% Yb^3+^. In this case, the number of the remaining photons relaxed to the lower energy levels than ^5^ F_3_ to give other emission is very small. Besides, double-exponential decay curves of Ho^3+^: ^5^ F_4_, ^5^S_2_ → ^5^I_8_ emission at 545 nm as well as ^5^ F_5_ → ^5^I_8_ emission at 651 nm with logarithmic coordinates are plotted with various Yb^3+^ concentrations in Figure 5b, c, and the highest resonant ET efficiencies for CR1(*η*_CR1_) and CR2(*η*_CR2_) are calculated to be 71% and 69%, respectively, which are also efficient. All the above results just indicate that the Yb^3+^: ^2^ F_5/2_ → ^2^ F_7/2_ emission should demonstrate stronger intensity and higher quenching concentration than those of Ho^3+^: ^5^I_6_ → ^5^I_8_ emission.

However, the NIR emission spectra in Figure 3 show distinct results. Based on these experimental results and combining the energy levels of Yb^3+^ and Ho^3+^, we brought up a novelty back ET process from Yb^3+^ to Ho^3+^ (Yb^3+^(^2^ F_5/2_) + Ho^3+^(^5^I_8_) → Yb^3+^(^2^ F_7/2_) + Ho^3+^(^5^I_6_) + *hν*) which may be occurring in the NIR QC system, as shown in the Figure 5d, since the back ET phenomenon widely exists in UC for Yb^3+^, Ho^3+^-doped materials [20,21]. This ET not only increases the Ho^3+^: ^5^ F_5_ → ^5^I_8_ emission intensity but also reduces the Yb^3+^: ^2^ F_5/2_ → ^2^ F_7/2_ emission intensity, resulting in the spectral features in Figure 3.

The QE is known to be defined as the ratio of the number of photons emitted to the number of photons that are absorbed. It can be concluded from Figure 5d that the extra photons emitted come only by CET and CR1 process for every phonon absorbed when excited at 450 nm. Therefore, supposing that all the excited Yb^3+^ and the residual excited Ho^3+^ decay radiatively, a modified calculation equation for the total QE (*η*_QE_) when excited at 450 nm can be theoretically expressed as follows according to [11]:

(4) $$\eta_{\text{QE}}=1+\eta_{\text{CET}}+\left(1-\eta_{\text{CET}}\right)\eta_{\text{CR}1}$$

where the last two terms stand for the extra QEs for CTE and CR1 processes, respectively. The total *η*_QE_ for BaGdF_5_: 15% Yb^3+^, 1% Ho^3+^ is calculated to 192% by this formulation. This so high QE partly results from the Ho^3+^^5^I_6_^5^I_8_ emission (1,180 nm), which is useless to enhance the efficiency of Si solar cells, since this emission does not match the spectral response of Si solar cells.

## Conclusions

Unlike the common situation that two emitting photons are from the acceptor Yb^3+^ ions, both the donor (Ho^3+^) and the acceptor (Yb^3+^) could emit NIR photons under blue light excitation. The visible emissions decrease with the introduction of the Yb^3+^ ions, while the NIR emissions at 980 nm and 1,180 nm are greatly enhanced. The quenching concentration of Ho^3+^ is higher than that of Yb^3+^. The fluorescence decay lifetimes of ^5^ F_3_ → ^5^I_8_, ^5^ F_4_, ^5^S_2_ → ^5^I_8_, and ^5^ F_5_ → ^5^I_8_ emissions of Ho^3+^ donors were recorded and calculated as a function of Yb^3+^ concentration. It could be concluded that NIR emissions are mainly through second- and first-order ET processes: Ho^3+^(^5^ F_3_) → 2Yb^3+^(^2^ F_5/2_) + *hν*, Ho^3+^(^5^S_2_) + Yb^3+^(^2^ F_7/2_) → Ho^3+^(^5^I_6_) + Yb^3+^(^2^ F_5/2_) + *hν* by spectra and decay curve analysis. The corresponding QE are calculated to be 192% in BaGdF_5_: 1% Ho^3+^, 15% Yb^3+^, so BaGdF_5_: Ho^3+^, Yb^3+^ nanoparticles could open up an approach in designing ultra-efficient photonic devices, for the application in low bandgap solar cells and thermo-photovoltaic energy conversion, etc.

## Competing interests

The authors declare that they have no competing interests.

## Authors’ contributions

LG participated in the design of the study, carried out the total experiments, and performed the result analysis, as well as drafted the manuscript. YhW participated in the design of the study, gave the theoretical and experimental guidance, and made the corrections of manuscript. JZ mainly helped in the experiments and measurements. YzW and PD gave the theoretical and experimental guidance and helped amend the manuscript. All authors read and approved the final manuscript.

## Authors’ information

LG, JZ, YzW, and PD are all Ph.D. candidates, and YhW is a Distinguished Young Scholar at the Department of Materials Science, School of Physical Science and Technology, Lanzhou University.
